# Occupational stress among Namibian diagnostic radiographers during the COVID-19 pandemic

**DOI:** 10.4102/hsag.v30i0.2823

**Published:** 2025-02-25

**Authors:** Festus Shidolo, Aladdin Speelman, Valdiela Daries

**Affiliations:** 1 Department of Medical Imaging and Therapeutic Sciences, Faculty of Health and Wellness Sciences, Cape Peninsula University of Technology, Cape Town, South Africa

**Keywords:** occupational stress, COVID-19, pandemic, diagnostic radiographers, radiology

## Abstract

**Background:**

The coronavirus disease 2019 (COVID-19) pandemic has significantly affected the health care sectors, causing stress among professionals such as diagnostic radiographers who helped fight this disease.

**Aim:**

This study explored occupational stress and stressors caused by the COVID-19 pandemic among diagnostic radiographers including coping strategies used and interventions that may be used to mitigate the effects of stress during future pandemics.

**Setting:**

The study was conducted among Namibian diagnostic radiographers.

**Methods:**

An electronic survey was used to collect data using a quantitative descriptive research approach. The Statistical Package for Social Sciences version 26 was used for statistical analysis.

**Results:**

Among the 90 diagnostic radiographers who responded to the survey, an increase in the workload, fear of contracting the virus and spreading it to others, were the main contributors of COVID-19 occupational-related stressors. Spending quality time with family and friends, developing hobbies and meditating and taking part in spiritual or religious activities were some coping strategies used to reduce stress. The introduction of incentives, social networking and provision of psychological support were preferred interventions that could mitigate the effects of occupational stress during similar pandemics.

**Conclusion:**

Diagnostic radiographers endured significant occupational stress because of the COVID-19 pandemic. Health care employers are encouraged to provide certain incentives and psychological support during similar pandemics as described in this article.

**Contribution:**

Radiography managers may consider the introduction of flexible working hours, provision of adequate staffing, participation and shared decision-making to mitigate stress during similar future pandemics.

## Introduction

The coronavirus disease 2019 (COVID-19) originated in December 2019 in Wuhan, China. The World Health Organization declared the COVID-19 outbreak as a global health emergency on 30 January 2020 (Akudjedu et al. 2020) and a global pandemic on 11 March 2020 (Tay et al. 2021). Diagnostic radiographers (‘henceforward referred to as radiographers’) and other health care professionals were at the forefront of fighting this disease (Lewis & Mulla 2021; Pereira et al. 2021). The demand for medical imaging to confirm a diagnosis and assess the progression of the COVID-19 related diseases was reported to be on the increase during the pandemic (Mc Fadden et al. 2022). Radiological imaging such as chest X-rays and Computed Tomography emerged as essential diagnostic investigations in managing COVID-19 infections (Flood, McFadden & Shepherd 2022; Ooi et al. 2021). Because of this increased demand for radiological imaging, radiographers were exposed to the virus and this potentially heightened their stress levels. As part of their clinical duties during this pandemic, radiographers experienced stress, exhaustion, separation from families, stigma and emotional pain because of the death of colleagues and patients (AlMulla 2020; Elshami et al. 2021). It was therefore conceivable that the COVID-19 pandemic presented additional occupational stress among radiographers (Akudjedu et al. 2020). The unsung radiographer heroes and heroines were worn out and exhausted as they responded to emergencies and answered to their humane call during this difficult time (Itani et al. 2021). Research focusing on health and particularly Radiography is generally understudied in Namibia. To the authors’ knowledge, no published studies could be identified describing COVID-19 occupational stress among radiographers in Namibia which suggested a knowledge gap in this area.

Namibia is a middle-income developing country with wide spread poverty. Like other countries, the COVID-19 pandemic caught the Namibian health care system unprepared. This study explored Namibian radiographers’ occupational stress and stressors caused by the COVID-19 pandemic, the coping strategies used and helped to identify interventions that can be used to mitigate the effects of stress during future pandemics.

### Research setting

The study was conducted in Namibia, a country located in South-West Africa. Namibia’s healthcare system consists of public and private sectors. The public health care services are managed by the Ministry of Health and Social Services. Radiographers in the public and private sectors provide essential radiographic imaging services. In Namibia, all practising radiographers are registered with the Health Professions Councils of Namibia (HPCNA), a statutory body upholding education and training standards for health care professionals (HPCNA 2024).

## Research methods and design

A quantitative, cross-sectional descriptive study using an electronic survey was conducted to collect data. Radiographers from both the public and private health facilities were invited to participate. Radiography assistants, students, lecturers, clinical instructors, radiation therapists, nuclear medicine technologists and ultrasonographers were excluded. During the study period, there were 207 registered radiographers in the country (HPCNA 2020). Given the small population of registered radiographers, a total population sampling was employed. Total population sampling is a purposive sampling technique considered suitable for small-sampled studies (Canonizado 2024). It is important to consider that if the sample size is small, it may not meticulously reflect the true characteristics of the population being studied (Kibuacha 2021). For these reasons, the whole radiographers population was purposively sampled to best answer the research question. No statistical formula was used to calculate the required sample size as the entire population was eligible to partake in the study. Considering that the sample size in the study was uncertain, our aim was to attain as many respondents as possible.

### Data collection

A self-developed electronic questionnaire using Google Forms was used for data collection. This questionnaire was developed based on related studies and adapted for the Namibian context (Akudjedu et al. 2020; Demirjiana et al. 2020; Elshami et al. 2021). The questions were designed in line with what the authors anecdotally deemed most relevant in capturing data to satisfy the study objectives. The questionnaire consisted of 19 questions with a mixture of close-ended, multiple-response options and Likert rating scales. A link to the electronic questionnaire was distributed mainly via a WhatsApp group for Namibian radiographers. In addition, electronic mails containing a link were further sent to private radiology heads of departments to distribute to their respective radiographers. Data were collected over a 4 week period.

The questionnaire was piloted on a sample of five radiographic assistants to test for validity and reliability. Respondents were asked for their opinion on understandability, clarity and flow of questions and the necessary modifications were made. Content reliability was measured by Cronbach’s alpha (*α*). Cronbach’s alpha ranges from zero (0) to one (1) whereby 0.7 is considered as a benchmark, with values near 0.7 and upward ideally acceptable (Frost 2023). Our questionnaire was found to be valid and reliable with Cronbach’s alpha measuring over 0.700 (0.700, 0,727, 0.791, 0.792, 0.796 and 0.846) indicating a good-to-excellent reliability. Respondents’ consent was presumed upon submission of the questionnaire.

### Data analysis

Survey data were transferred from Google forms into the Statistical Package for the Social Sciences version 26.0 (International Business Machines Corporation, Armonk, New York, United States). The quantitative variables were presented in percentage format, while the Chi-square test (*χ*^2^) was used to assess whether there was any association among categorical variables; for example, if a relationship existed between fear of contracting or spreading COVID-19 and the age category among others. A *p*-value of ≤ 0.05 was considered statistically significant. Responses to the open-ended questions were analysed and reported as they were, with no specific thematic analysis applied. This was as per Gell (2019) who stated that providing the entire list of texts was another acceptable approach of presenting open-ended responses.

### Ethical considerations

The study was approved by the Research Ethics Committee, Faculty of Health and Wellness Sciences of the Cape Peninsula University of Technology (study approval number: CPUT/HWS-REC 2022/H5). All applicable ethical standards were upheld during data collection in accordance with the Helsinki declaration (1964) and later amendments.

## Results

A total of 90 radiographers out of 207 registered with the HPCNA in 2020 completed the survey resulting in a 43% response rate. Not all questions were answered by the respondents, resulting in some total number of respondents not adding to 90. The majority of respondents were females (84%) (*n* = 72/86) compared to 16% males (*n* = 14/86). Respondents who identified themselves as ordinary radiographers were 74% (*n* = 64/87) while 26% (*n* = 23/87) were radiographers in managerial roles. The majority of respondents (41%) (*n* = 36/88) were within the age group 20–29 years and the least (6%) (*n* = 5/88) within the age group of 50–59 years. More respondents (55%) (*n* = 48/88) were from the private sector, compared to those from the public sector (45%) (*n* = 40/88).

**FIGURE 1:**
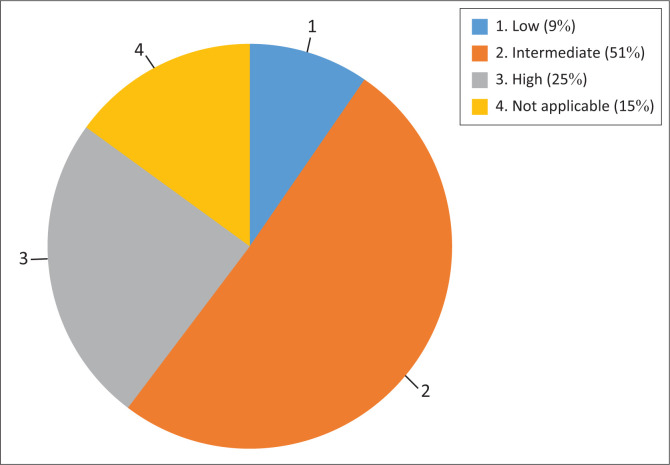
Level of occupational stress because of COVID-19.

From the cross-tabulation, it was found that stress for fear of contracting COVID-19 at work was higher than expected in respondents from public health care facilities (32 counts out of 24.7 expected) compared to respondents from private facilities (23 counts out of 30.3 expected). There was a statistically significant association between respondents working at public and private healthcare facilities and their fear of contracting COVID-19 caused them stress at work (*χ*^2^ = 10.795^a^, *p* = 0.005). The current study showed that there was no statistically significant association between the age category of the respondents and their tendency to be fearful of contracting COVID-19 at the workplace (*χ*^*2*^ = 4.724^a^, *p* = 0.858). Similarly, the Chi-square test found no significant statistical association between the age category of the respondents and being fearful of spreading COVID-19 (*χ*^*2*^ = 3.839^a^, *p* = 0.922). Respondents were further asked to rate their occupational stress during the COVID-19 pandemic. Their responses are presented in Figure 1.

### Stressors associated with COVID-19 pandemic

**FIGURE 2:**
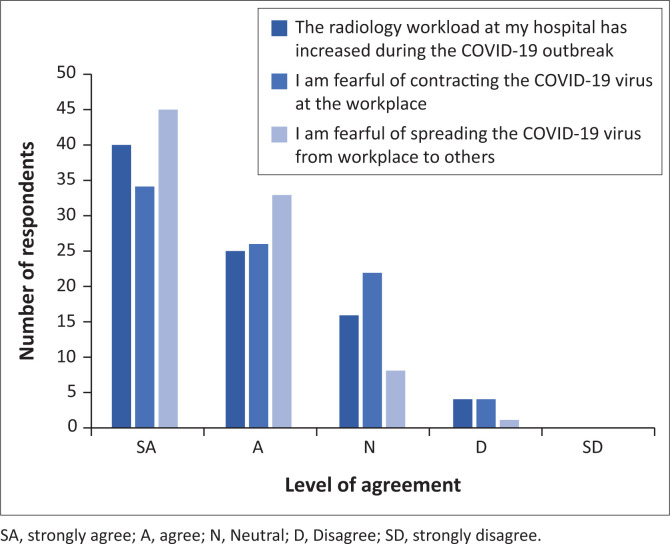
Top-three COVID-19 pandemic stress factors identified by respondents.

The respondents were provided with a table highlighting stressors associated with the COVID-19 pandemic as identified in literature. Respondents were asked to indicate which of the listed stressors caused them stress at work by choosing the level of agreement with the statement on the Likert scale provided. From the responses received, increased workload, fear of contracting the COVID-19 virus from the workplace and fear of spreading it to others were the top three stressors identified by respondents (see Figure 2).

Respondents were also asked to indicate other factors that caused them stress during the COVID-19 pandemic. These responses are listed in Table 1.

**TABLE 1:** Other factors that caused stress among respondents.

Statement	SA		A		N		D		SD		Total	
	*n*	%	*n*	%	*n*	%	*n*	%	*n*	%	*n*	%
**PPE:** Personal protective equipment was available at my workplace during the COVID-19 outbreak	33	38	33	38	10	12	9	10	2	2	87†	100
**Staff:** There is enough staff complement at my work to deal with the COVID-19 pandemic workload	12	14	36	41	25	29	9	10	5	6	87†	100
**Preparedness:** My work has prepared me to deal with COVID-19 patients during the outbreak	19	22	37	43	21	24	10	11	0	0	87†	100
**Cleaning material:** Supply as an infection control measure is available at my workplace	33	38	40	46	7	8	6	7	1	1	87†	100
**Disinfectant material:** Supply as an infection control measure is available at my workplace	37	42	38	44	7	8	4	5	1	1	87†	100
**Physical distancing:** My workplace practises physical distancing as a preventative tool against contracting the COVID-19 virus	21	24	39	45	12	14	10	11	5	6	87†	100
**Adaptation:** I find it difficult adapting to sudden new changes in the workplace because of the COVID-19 pandemic	14	16	32	37	27	31	10	11	4	5	87†	100
**Support:** I am receiving support from management to keep me motivated during the COVID-19 pandemic	9	10	31	36	27	31	16	18	4	5	87†	100
**Appreciation:** I feel I am appreciated for the work I am doing during the COVID-19 pandemic	13	15	24	27	25	29	11	13	14	16	87†	100

Note: Values in this table represent the actual number of observations (*n*).

SA, strongly agree; A, agree; N, Neutral; D, Disagree; SD, strongly disagree.

† , Questions not answered by all respondents.

### Coping with COVID-19 occupational stress

**FIGURE 3:**
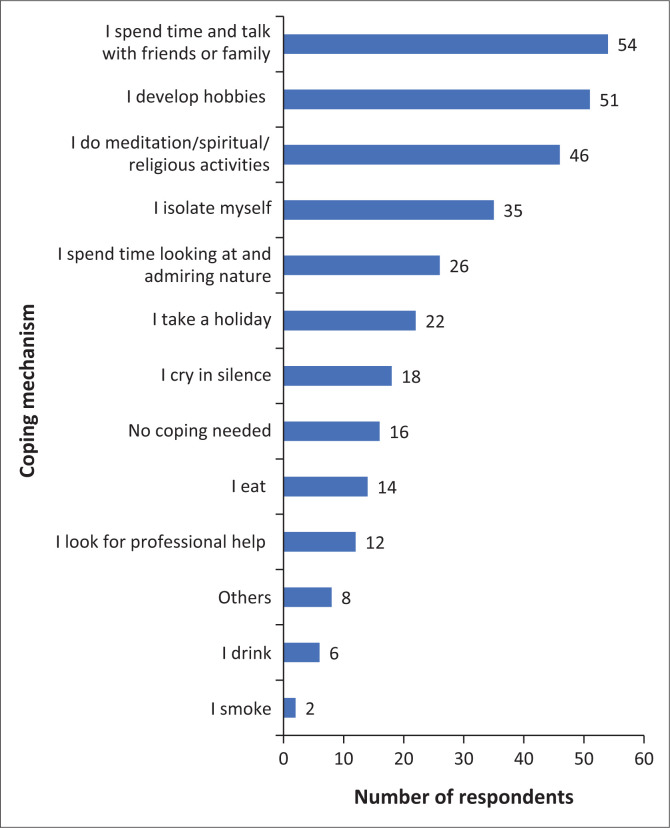
Coping strategies used by respondents.

Respondents were asked to indicate the methods used for coping with stress related to the COVID-19 pandemic. Respondents indicated that spending quality time with and talking to family or friends (60%) (*n* = 54/90) and developing a hobby (57%) (*n* = 51/90) were their preferred coping strategies. Meditation and taking part in spiritual and religious activities (51%) (46/90) and isolating themselves (39%) (35/90) were the third and fourth most preferred coping strategies respectively. Drinking alcohol (7%) (*n* = 6/90) and smoking (2%) (*n* = 2/90) were the least stated options. Eighteen per cent (*n* = 16/90) of the respondents recorded no need for any coping strategies (refer to Figure 3).

### Suggestions and general comments

The last two questions of the survey were open-ended aimed at eliciting suggestions on how COVID-19-related occupational stress could be better managed by the employer and allowed general comments relative to the COVID-19 pandemic. Suggestions made by different respondents included *‘better working hours’, ‘better compensation’, ‘psychological support’, ‘moral and faith support’, ‘taking radiographers opinions and suggestions into consideration’, ‘trainings and workshops’, ‘… meetings to discuss issues affecting radiographers.’, ‘… provide PPE’, ‘Emphasis is always placed on the doctors and nurses leaving out radiographers …’, ‘appreciation’, ‘hire more staff’, ‘recognise radiographers as frontline staff’.*

## Discussion

The first objective of the study assessed radiographers’ occupational stress and stressors caused by the COVID-19 pandemic. The most significant stressors among radiographers were increased workload, fear of contracting the COVID-19 virus and spreading it to others. The fear regarding contracting the COVID-19 virus was in keeping with findings from other studies (Akudjedu et al. 2020; Naylor et al. 2021; Van de Venter et al. 2021). In one other study, fear was reported to have caused health care professionals to sleep in their cars to protect their families (Khan et al. 2020).

Our study revealed that the respondents at public health care facilities were more fearful of contracting the COVID-19 virus at the workplace compared to their counterparts in private facilities. This finding was higher than anticipated and was calculated to be statistically significant. The study revealed that no statistically significant association existed between the respondents’ age category and being fearful of contracting the COVID-19 virus at the workplace and spreading it to others. No statistically significant association was found between the respondents’ age category and fear of spreading the COVID-19 virus.

In the current study, the increased workload was found to intensify the level of stress among radiographers, a finding also reported by Ibrahim, Akib and Jaffar (2021). The increased workload in radiology departments during the pandemic was reportedly because of radiographers having to work long hours without a break time (Adesi, Kwadwo & Kab 2015).

The second objective explored coping strategies that radiographers used to manage occupational stress during the COVID-19 pandemic. A large number of radiographers in the current study endured occupational stress both prior to and during the COVID-19 pandemic. The coping strategies mostly preferred by radiographers were spending quality time with and talking to either a friend or a family member, developing hobbies and partaking in sport, exercising, watching television, reading, cooking, listening to music, meditating, spiritual and religious practices as well as self-isolation. Radiographers in this study used exercising to cope with pandemic-related stress which aligned with that of Knapp et al. (2022). Another study by Shechter et al. (2020) also found physical activity and spiritual practices to be the most common stress-reduction activities undertaken by health care professionals during the COVID-19 pandemic.

Regardless of the difference in coping measures, radiographers should utilise whichever coping method best works for them. Radiographers need to have social contact between themselves and their family and friends to discuss any conflicting issues they are experiencing at work (Knapp et al. 2022; Mudenda et al. 2022). Ashong et al. (2016) claimed that applying coping strategies regularly and frequently could lower stress levels. The authors believe that pastoral and religious support should be made available to radiographers promptly whenever needed.

The current study also found that a very small portion of respondents resorted to eating, drinking alcohol and smoking as their coping strategies. Alcohol use as a coping mechanism during the COVID-19 pandemic was also described in another study (Reilly et al. 2021). While it is common for some health care professionals (including radiographers) to use substances during stressful times to help them cope with the COVID-19 pandemic, health care professionals should be warned of the danger associated with using such coping strategies (Reilly et al. 2021).

The third objective of the study was to identify and describe workplace interventions that can be used to mitigate the effects of stress among radiographers during future pandemics. The top three interventions cited by respondents in this study included the introduction of incentives, social networking and provision of psychological support. One study showed that an important reported fear of seeking psychological support was stigmatisation (Khan et al. 2020). Health care professionals in another study reported that accessing emotional and psychological support during the COVID-19 pandemic might be embarrassing should it be revealed that they were seeking such support (Feeley et al. 2021).

Other suggestions expressed by respondents from our study regarding how future pandemics can be managed by their employers included provision of training and workshops, monetary incentives, hiring of more radiographers and flexible working hours. Respondents further desired platforms where they can directly engage management and discuss matters of concern for their opinions and suggestions to be considered during decision-making. The latter suggestion implied that respondents did not feel involved in the general decision-making processes. Management should therefore avail such platforms where respondents can express their views and participate in decision-making.

One of the most important points raised under general comments was that radiographers wanted recognition as frontline workers just like other health care professionals such as nurses, doctors, pharmacists and others. Radiographers indicated that they were involved in many of the critical diagnostic activities related to the COVID-19 pandemic but were not regarded and treated equally compared to other professionals whom they thought were given more privileges by hospital managers. Radiography managers might consider remedies aimed at restoring the sense of feeling being valued (Prasad et al. 2021).

### Strengths and limitations

Our study focused on the stressors Namibian radiographers faced at their workplaces during the COVID-19 pandemic. To the authors’ knowledge, this study is the first of its kind to be conducted among Namibian radiographers. Hence, the study provided unique information about their experiences concerning COVID-19-related occupational stress.

The study had some noteworthy limitations. With the use of the electronic survey, radiographers with no smart cellular phones, or access to personal computers and those deep in remote areas with no internet coverage did not have a fair chance to participate. Consequently, the experiences of radiographers working in such smaller hospitals and remote parts of the country were potentially excluded thus preventing the authors from gaining their valuable insights too.

The data were collected 2 years after the onset and peak of the COVID-19 pandemic; therefore, radiographers may have developed resilience towards the pandemic. Additionally because of this time-lapse, recall bias could have distorted their memory. While the whole radiographer population was purposively sampled, the limited response rate of 43% recorded does not represent the opinions and experiences of the sample population and the findings therefore cannot be generalised. Our study did not investigate whether there was a discrepancy in acquisition of personal protective equipment and access to resources between the private and public institutions during the pandemic. This can be explored by future studies.

### Implications of this study

The findings of this study were based on radiographers and could not be generalised to the entire Radiography workforce, including those employed in other disciplines. Future studies may explore how radiographers in radiotherapy, ultrasonography and nuclear medicine were affected. Future studies can also employ a larger sample size among radiographers. This study did not ascertain how many radiographers made use of counselling and support progammes during the pandemic. Future studies can therefore explore this aspect in future pandemics.

## Conclusion

Stress is pervasive in the Radiography profession and Namibian radiographers faced similar COVID-19 occupational-related stress experienced by colleagues globally. Overall, the increased workload and fear of both contracting and spreading the virus were rated as high sources of stress among radiographers. Despite the stressful situations in the workplace, different coping methods enabled Namibian radiographers to withstand the effects of COVID-19 occupational-related stress. The study recognised the important role played by families and friends in offering immediate support to the radiographers during this pandemic. The introduction of incentives, building a strong social network and provision of psychological support systems for radiographers are additional methods that can be used to alleviate stress during such pandemics.
